# Evolution of Cooperative Cross-Feeding Could Be Less Challenging Than Originally Thought

**DOI:** 10.1371/journal.pone.0014121

**Published:** 2010-11-29

**Authors:** Sylvie Estrela, Ivana Gudelj

**Affiliations:** Department of Mathematics, Imperial College London, London, United Kingdom; University of Bristol, United Kingdom

## Abstract

The act of cross-feeding whereby unrelated species exchange nutrients is a common feature of microbial interactions and could be considered a form of reciprocal altruism or reciprocal cooperation. Past theoretical work suggests that the evolution of cooperative cross-feeding in nature may be more challenging than for other types of cooperation. Here we re-evaluate a mathematical model used previously to study persistence of cross-feeding and conclude that the maintenance of cross-feeding interactions could be favoured for a larger parameter ranges than formerly observed. Strikingly, we also find that large populations of cross-feeders are not necessarily vulnerable to extinction from an initially small number of cheats who receive the benefit of cross-feeding but do not reciprocate in this cooperative interaction. This could explain the widespread cooperative cross-feeding observed in natural populations.

## Introduction

Cross-feeding between unrelated species, termed syntrophy, is the ability of one organism to use metabolites excreted by another organism [1]. When this interaction involves a reciprocal exchange between the partners as a cooperative behaviour and not merely an exchange of waste products as a result of a selfish act, cross-feeding can be considered a mutualistic act known as reciprocal altruism [2] or reciprocal cooperation [3], [4]. Such behaviour is common in the microbial world [4]–[8] and is of a fundamental importance to our understanding of microbial communities and their impact on the environment. A remarkable example can be found in the association between archaea and bacteria that couple methane oxidation with sulfate reduction, respectively. This syntrophic association has been estimated to involve the consumption of more that 80% of the ocean methane flux and is an important process needed to reduce the emissions of the green house gas methane from the ocean into the atmosphere [9]–[11]. Syntrophic interactions are also known to play a key role in the degradation of xenobiotic compounds [12] which is crucial for the minimization of surface and ground water contamination by pesticides. Other examples of syntrophy include interactions between fermentative bacteria and methanogenic archeon [13]; methanogens and ethanol fermenters [14], [15] and between green-sulphur bacteria and the β-proteobacteria [16].

While the importance of cross-feeding syntrophy is clear, what is less clear is how can a group of individuals who engage in such form of cooperative behaviour resist invasion by cheats who do not pay the cost of cooperation but reap the reward? A model exploring the conditions favouring the origin of cooperative cross-feeding between two microbial species was recently proposed by [17]. There the authors uncover some unintuitive constraints, namely that the benefit of cooperative cross-feeding applies only in the range of intermediate cell densities and is more easily selected when the cost of cross-feeding to the donor is low per benefit to the recipient and when the recipient already provides a large cross-feeding benefit to the donor. This finding is contingent on the existence of a trade-off between the cost to cooperators of performing an altruistic act and the benefit to the recipients towards whom the cooperation is directed. Such trade-off arises naturally from the definition of a cooperative act because a cross-feeding cooperative individual sacrifices its intrinsic growth to benefit other species by facilitating their ability to grow. The authors also find that large populations of cooperative cross-feeders are vulnerable to exploiting genotypes (or cheats) who share the cross-feeding resources but do not reciprocate in the cross-feeding themselves.

In this paper we revisit the model presented in [17] and highlight a number of parameter regimes that tend to increase the window in which cooperation is favoured. Contrary to [17] we find that large populations of cross-feeders are not easily taken over and replaced by a small number of cheats. This result relies on the assumption that all types have the same carrying capacity. Subsequently we present an alternative evolutionary model that relaxes the assumption of equal carrying capacities and again show that replacement of cooperators by cheats is not the most common outcome of evolution.

## Results

### The mathematical model

In [17] the authors propose the following model of cross-feeding. Consider a spatially heterogeneous environment containing two separate local patches. Each patch contains a pair of clonal microbial populations interacting by cross-feeding in the following way. Patch 1 contains genotypes X and Y engaged in a cross-feeding syntrophy with X cross-feeding Y and Y cross-feeding X. Patch 2 contains genotypes X_c_ and Y whereby X_c_ receives a cross-feeding benefit from Y but does not reciprocate in the cross-feeding. Population dynamics of each patch are subsequently modeled as follows:

### Patch 1 model

Let *X(t)* and *Y(t)* denote densities of genotypes X and Y respectively, at time *t*. The rate of expansion of the X population is governed by:

1. *an intrinsic ability to grow* denoted by *r_x_*;
2. *the per capita level of cross-feeding* described by 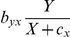 where *b_yx_* represents a benefit to X per individual of type Y and *c_x_* represents a damping constant that sets the cross-feeding resource proportional to *Y* when *X* is vanishingly small;
3. *crowding* implemented through a total carrying capacity *K* of the two microbial types.

Applying the same population expansion rules to type Y leads to the following system of equations
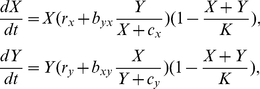
(1)where *r_y_* denotes the growth constant for the population of type Y, *b_xy_* represents a benefit of cross-feeding to Y per individual of type X with the assumption that *b_xy_ = b_yx_*. The parameter *c_y_* denotes a damping constant that sets the cross-feeding resource proportional to *X* when *Y* is vanishingly small.

### Patch 2 model

Let *X_c_(t)* denote the density of genotype X_c_ at time *t*. The model (1) can be adapted to describe interactions between X_c_ and Y as follows
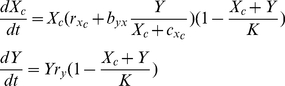
(2)where 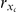 denotes the growth term of non cross-feeder X_c_ with 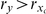 while 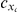 denotes the cross-feeding damping constant defined in a similar way as *c_x_* in the model (1).

**Figure 1:**
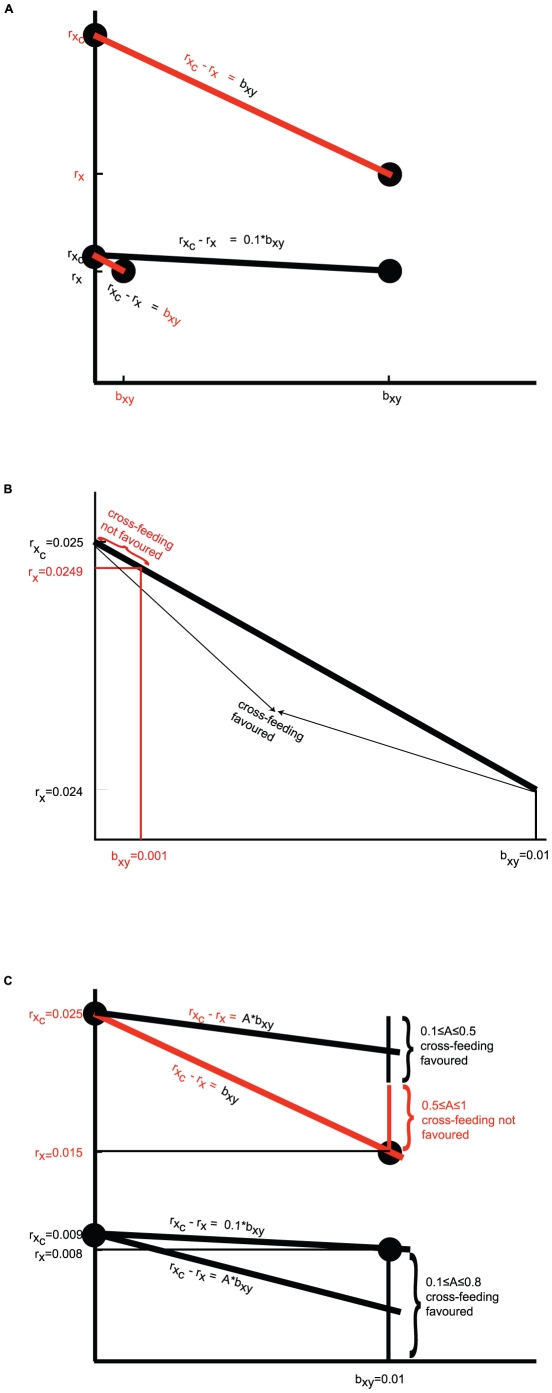
Trade-off between the cost of cooperation and the benefit to the recipient determines cross-feeding success. Whether cross-feeding is favoured at intermediate densities depends on: **A**. the slope of the trade-off function with cross-feeding more easily selected for shallow slopes; **B**. the values of the cost (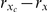) and the benefit (*b_xy_*) of cross-feeding with cross-feeding more easily selected for high 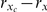 and *b_xy_*; **C**. the value of the intrinsic growth parameters (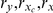) with cross-feeding more easily selected for low 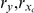 and *r_x_*. Throughout the figure black lines denote cases where cross-feeding is favoured while red lines denote cases where cross-feeding is not favoured.

X_c_ can be viewed as a non-cooperative (or cheating) genotype. By definition a cooperative trait carries a cost to cooperator of performing an altruistic act while providing a benefit to the recipient towards whom the cooperation is directed. Just as in [17] we assume the existence of a trade-off between *r_x_* and *b_xy_* (as well as between *r_y_* and *b_yx_*) which means that a cross-feeding individual of a given type sacrifices its own growth to facilitate the growth of another type. Therefore, comparing model (1) and (2) we note that 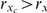 because X_c_ does not pay a cost of cooperation and that 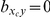 as X_c_ does not provide a cross-feeding benefit to Y and hence there is no bidirectional cross-feeding (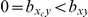). This forms a part of the cost/benefit trade-off and is illustrated in Figure 1.

### The dynamics of model (1)

The cross-feeding model (1) has the following steady states
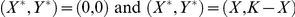
where 0*≤X≤K*.

The eigenvalues of the linearised system (1) around the zero state are *λ_1_ = r_x_/r_y_* and *λ_2_* = 1 and since both *λ_1_*>0 and *λ_2_*>0 we conclude that (0,0) is an unstable steady state.

Therefore a small population (*X*(0),*Y*(0)) = (*ε_1_,ε_2_*) with *ε_1_* and *ε_2_* denoting positive constants near zero, will initially grow away from the zero steady state according to the following equation:
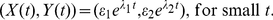
(3)Subsequently the solution of (2) will approach one of the infinitely many steady states (*X*,*K−X*) situated on the line segment *Y = K−X*. Which steady state it converges to cannot be determined with classical linearization techniques and will depend on the initial population sizes *ε_1_* and *ε_2_*.

### The dynamics of model (2)

Similarly the model (2) has the following steady states
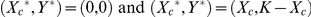
where 0≤*X_c_*≤*K*.

The eigenvalues of the linearised model (2) around the zero steady state are 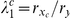 and *λ_2_ = 1* and since both 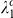
>0 and *λ_2_*>0 we conclude that (0,0) is an unstable steady state.

Therefore a small population (*X_c_*(0),*Y*(0)) = (*ε_1_*,*ε_2_*) will initially grow away from the zero steady state according to the following equation
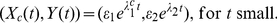
(4)Subsequently the solution of (2) will approach one of the infinitely many steady states (*X_c_*,*K−X_c_*) situated on the line segment *Y = K−X_c_*. As for model (1), which steady state it converges to will depend on the initial population sizes *ε_1_* and *ε_2_*.

### Comparing the dynamics of models (1) and (2)

As in [17] the success of the non-cross feeding strategy is examined by comparing the cross-feeding genotype to the non cross-feeding genotype across the two patches. In other words starting with the same initial population densities (*X*(0),*Y*(0)) = (*ε_1_*,*ε_2_*) and (*X_c_*(0),*Y*(0)) = (*ε_1_*,*ε_2_*) in patch 1 and patch 2 respectively, the *X*(*t*) component of the solution of (1) representing densities of the cross-feeding strategy X is compared with the *X_c_*(*t*) component of the solution of (2) representing the density of the non cross-feeding strategy X_c_.

From (3) and (4) it follows that
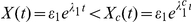
for some small time *t*. Therefore as found in [17], at low population densities X_c_ always does better than X because 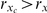 and therefore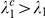. This means that at low densities the cost of cooperation is not compensated by the benefit of cross-feeding.

Whether there exist a time interval for which the cross-feeding genotype does better than the non-cross feeding genotype (*X*(*t*)>*X_c_*(*t*)) depends on a range of assumptions regarding the nature of the trade-off between the cost of cooperation and the benefit to the recipient, the initial population densities as well as the values of the intrinsic growth rates and/or the benefit of cross-feeding. For growth at intermediate densities the study presented in [17] generates the following results:

- BH1: When *b_yx_*>0, selection always favours reciprocal cross-feeding from X to Y when *r_x_≤r_y_*.
- BH2: Trade-offs with big gains in *b_xy_* per decline in *r_x_* enhance evolution of cooperation.
- BH3: Large *b_yx_* enhance the evolution of reciprocity in the other direction from X to Y.

**Figure 2:**
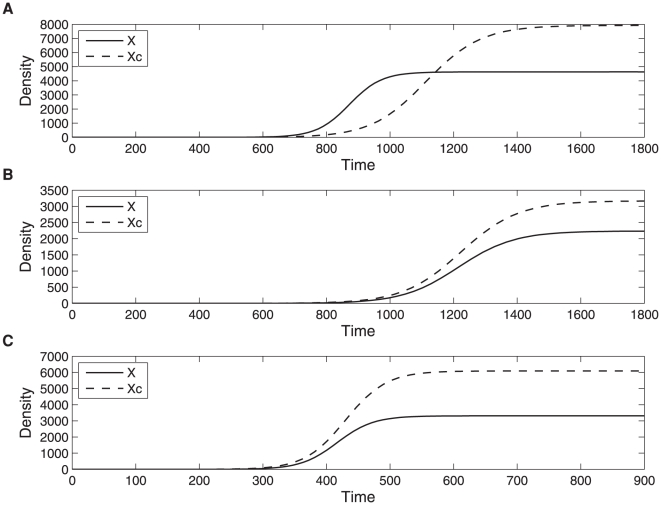
Simulation of two-species population growth for the model (1) and model (2). In the case of model (1) type X and Y cross-feed each other and in the case of model (2) X_c_ doesn't cross-feed Y but Y cross-feeds X_c_. Here we plot *X*(*t*) solution of (1) (full line) together with *X_c_*(*t*) solution of (2) (dashed line) with **A**. *r_y_* = 0.011, *r_x_* = 0.008, 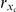 = 0.009, *b_xy_* = *b_yx_* = 0.01; **B**. *r_y_ = 0.011*, *r_x_* = 0.008, 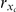 = 0.009, *b_xy_* = *b_yx_* = 0.001; **C**. *r_y_* = 0.03, *r_x_* = 0.015, 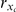 = 0.025, *b_xy_* = *b_yx_* = 0.01. For both simulations of model (1) and (2) and in all three cases presented here *K* = 10000, 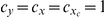 and *ε_1_* = *ε_2_* = 0.01.

The above results have been generated by approximating non-linear dynamics with a linear model. In this paper we revisit BH1-BH3 for the non-linear models (1) and (2) assuming that each model has the same initial population densities of both genotypes (*ε_1_* = *ε_2_*). Our study shows that BH1 does not hold in general. As illustrated in [17], we find that cross-feeding from X to Y is favoured if the slope of the trade-off curve satisfies 
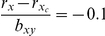
, in other words if the cost of cross-feeding is 10% of the value of the benefit of cross-feeding, and if *b_xy_* is sufficiently large (Figure 1A). In that case the cross-feeder X outgrows the non cross-feeder X_c_ for some intermediate time between the initial exponential growth and the final stationary phase (Figure 2A). However we find that changing the slope of the trade-off function has a profound effect on the above outcome. In particular we consider the case where the slope of the trade-off function is changing from shallow (−0.1) to steep (−1). Decreasing the slope can be achieved either by lowering the benefit of cross-feeding (*b_xy_*) or by increasing the cost of cooperation (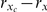) (see Figure 1A). In both cases we find that the cross-feeders never outgrow the non cross-feeders i.e *X*(*t*)<*X_c_*(*t*) all *t*>0 (Figure 2B,C). Note that in the case where *b_xy_* has been decreased (Figure 2B) the parameter *b_yx_* was also altered so that *b_xy_ = b_yx_*. Also note that in the case where the cost of cooperation has been increased (Figure 2C) the intrinsic growth rate of the Y genotype, *r_y_*, is modified so that the assumption 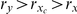 is upheld.

Whether the cross-feeding is favoured at intermediate densities is not solely determined by the slope of the trade-off function. For example retaining the shallow slope of −0.1 but changing the benefit of cooperation indicates that a small benefit (and therefore a small cost) of cross-feeding is less likely to favour the cross-feeding (Figure 1B). While this finding again contradicts BH1 it is in agreement with the result BH3 given that we assume that *b_xy_ = b_yx_*.

The result BH2 states that shallow trade-offs enhance the evolution of cooperation. While our findings agree with BH2 our results show that depending on the *r* and *b* parameter values, steep trade-offs can also promote the evolution of cooperation. For example the lower the values of 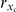 and *r_x_* (and by definition *r_y_*), the steeper the angle of the trade-off for which the cross-feeding is favoured at intermediate densities (Figure 1C). Keeping *b_xy_* fixed Figure 1C illustrates that when 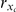 = 0.009 and *r_x_* = 0.008 the cross-feeding is favoured for trade-off slopes satisfying 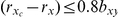. However, when 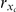 = 0.025 and *r_x_* = 0.015 cross-feeding is favoured for less steeper slopes 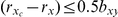. Note that when 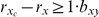 the cross-feeding is never favoured.

**Figure 3:**
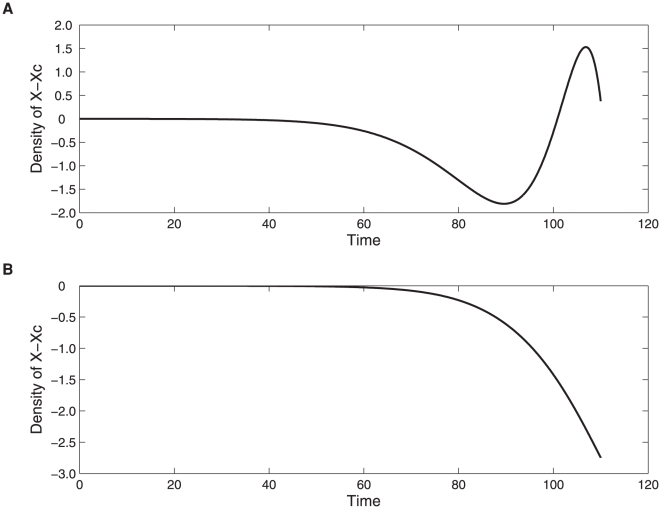
Initial population densities influence whether the cross-feeding will be favoured. For both simulations of model (1) and (2) *r_y_* = 0.11, *r_x_* = 0.088, 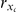 = 0.09, *b_xy_* = *b_yx_* = 0.01, *K* = 10000 and 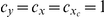. **A**. cross-feeding is favoured for initial conditions *ε_1_* = *ε_2_* = 0.01; **B**. cross-feeding is not favoured for initial conditions *ε_1_* = *ε_2_* = 0.001.

Reducing the initial population densities for both models (1) and (2) can lead to a dramatic change in the outcome from cross-feeding being favoured at intermediate densities (Figure 3A) to cross-feeders never outgrowing the non cross-feeders (Figure 3B). Similar results have been observed in [17].

We also note that changing the slope of the trade-off relationship has an impact on the final population densities. For example comparing the outcomes of Figures 2A and 2B it can be seen that decreasing the benefit of cross-feeding leads to lower final population sizes of both X and X_c_ genotypes. This could be explained in the following way. Decreasing the benefit of cross-feeding lowers the impact of cross-feeding on population growth and therefore growth of different genotypes is dominated by their intrinsic ability to grow. Given that 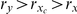 the Y genotype dominates the dynamics of both model (1) and (2) resulting in a smaller final population sizes of both X and X_c_. Similarly, by comparing the outcomes of Figure 2A and 2C it can be seen that an increase in the cost of cooperation also results in lower final population sizes of both X and X_c_. In this case an increase in the cost of cooperation was achieved by increasing 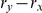 and 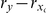 so that again the genotype Y dominated the dynamics of both model (1) and (2) resulting in a smaller final population sizes of both X and X_c_.

### Evolutionary dynamics

#### Competition between cheats and cooperators

So far we have been considering a scenario where pairs of interacting microbial genotypes engaging in different levels of cross-feeding grow in two isolated patches or colonies [17]. One could envisage a situation where at some point the populations will become large enough so that other types could migrate or could arise by mutation. This immediately raises the following question. What would happen to the equilibrium dynamics in patch (1) if a small amount of a cheating genotype X_c_ is introduced either through migration from patch 2 or through mutation in genotype X? To answer this question model (1) can be adapted as in [17] to include an equation for the cheating genotype X_c_:
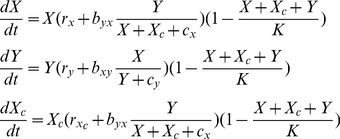
(5)We are interested in the dynamics of (5) given the initial conditions (*X*(0),*Y*(0),*X_c_*(0)) = (X^*^,Y^*^,ε), where (X^*^,Y^*^) is a non-zero steady state of model (1) and *ε* is a small constant. Such initial conditions denote the fact that a small population of non-cross feeding cheats has been introduced into patch 1 after its resident population has reached an ecological equilibrium.

**Figure 4:**
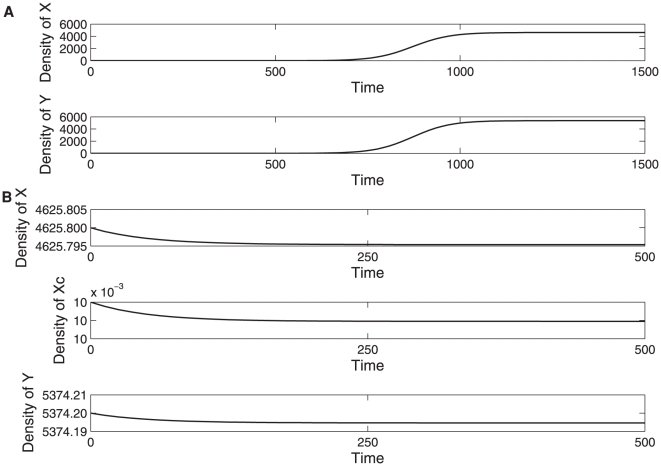
Evolutionary dynamics where mutants do not invade. **A**. Numerical simulations of the model (1) with *r_y_* = 0.011, *r_x_* = 0.008, *b_xy_* = *b_yx_* = 0.01, *K* = 10000 and 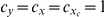. The figure shows an initial population *X*(0) = *Y*(0) = 0.01 converging to a steady state (X^*^,Y^*^). **B**. Numerical simulations of the model (5) where a small amount of non-cross feeder (*X_c_*(0) = 0.01), is introduced into the steady state population (X^*^,Y^*^). The figure shows that the cross-feeder X is not vulnerable to invasion by non-cross feeder X_c_. In addition to the above parameters 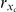 = 0.009.

Apart from the zero steady state the model (5) has infinitely many steady states satisfying the equation *X+Y+X_c_ = K*. As with models (1) and (2) the local stability of these steady states cannot be determined from simple linearization techniques. Numerical simulations indicate that for an initial condition (X^*^,Y^*^,ε) the model (5) will converge to a steady state (X^*^−δ_1_,Y^*^−δ_2_,δ_1_+δ_2_) where *δ_1_* and *δ_2_* are small constants (Figure 4). This means that once established the cooperator genotype is not necessarily vulnerable to exploitation by the cheating genotype. Instead the cheat remains in the population but at low levels, close to the initial value *ε*.

A similar observation can be made for the case where a small amount of cooperator genotype is introduced into patch 2 whose resident genotypes have reached an ecological equilibrium.

#### Evolution of cooperation

The competition model (5) assumes that all interacting types have the same carrying capacity, which in practice might not always be the case. In fact a cross-feeding between unrelated species often involves organisms that specialize on different resources. One such example is the interactions between two mutant strains

*Escherichia coli* and *Salmonella enterica ser. Typhimurium* described in [18]. Both strains were grown in lactose but *Salmonella* is not able to utilize lactose as an energy resource and instead uses a metabolite (acetate) excreted by *E.coli*. On the other hand, *E.coli* can only degrade lactose in the presence of the amino acid methionine, which is synthesized by *Salmonella* but not by *E.coli*.

Motivated by [18] we alter the assumptions in (5) in order to explore general conditions for the evolution of cooperative cross-feeding. We begin by assuming that there is no interspecific competition for resources between the two cross-feeding types X and Y. This assumption is motivated by the fact that *E.coli* and *Salmonella enterica ser Typhimurium* do not utilize the same limiting nutrient as energy source and therefore do not compete for the same resource. For simplicity we also assume that the benefit of cross-feeding is simply proportional to the density of the individuals of the type providing nutrients. Therefore the cross-feeding interactions between X and Y can now be written as:
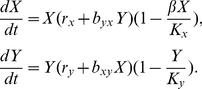
(6)where *β* is the parameter describing the intraspecific competition amongst individuals of type X while *K_x_* and *K_y_* denote carrying capacities of X and Y respectively. The above system (6) has a trivial (0,0), two semi-trivial (*K_x_/β*,0), (0, *K_y_*) and the non-trivial steady state (*K_x_/β,K_y_*). While the trivial and both semi-trivial steady states are unstable, the non-trivial steady state is stable (see Text S1).

We choose *b_xy_*, the benefit of cross-feeding to Y per individual X, as the evolving trait belonging to a one-dimensional phenotypic trait space [0, *b_xymax_*]. This phenotype can be viewed as an investment made by X into cooperation so that individuals with *b_xy_* = 0 do not invest into cooperation while individuals with *b_xy_* = *b_xymax_* invest maximally into cooperation. We assume that there will always be a biologically feasible maximum to any investment.

We now consider the effect of adding a mutant type X_m_ with phenotypic characteristic *b_xym_* to the system (6) that is at the non-trivial steady state (*K_x_/β,K_y_*). The evolution of the benefit of cross-feeding to Y per individual X (*b_xy_*) is governed by the following three trade-offs:

1. The trade-off between investment into cooperation (*b_xy_*) and an intrinsic ability to grow (*r_x_*) is denoted by *r_x_* = *f*(*b_xy_*), which is a decreasing function of *b_xy_*.
2. We also assume an asymmetric competition between the resident type X and a mutant type X_m_, whereby increased investment into cooperation results in an increased competitive ability. This can in part be justified by the inevitable existence of structure with a given environment. For example *Salmonella* strains that produce large amount of methionine could have a larger amount of acetate in their neighbourhood (created by *E.coli* through cross-feeding) than the *Salmonella* types producing less methionine. Therefore we define a function *β*(*b_xy_*−*b_xym_*) describing the effect of the mutant strategy *b_xym_* on the resident strategy *b_xy_* which is a decreasing function of *b_xy_*−*b_xym_*.
3. Finally we assume the existence of a trade-off between the investment into cooperation and the carrying capacity *K_x_*, where the carrying capacity is now a decreasing function of *b_xy_*, denoted by *K*(*b_xy_*). This assumption is motivated by the known inhibitory properties of methionine [19] so that an increased investment into cooperation leads to the over production of methionine which in turn leads to a reduction in the carrying capacity of the cooperating producer.

The equations for the new (mutated) system are given by:
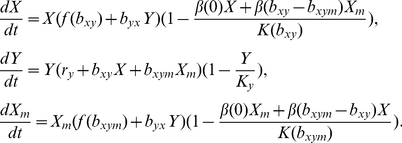
(7)The fitness of the invading mutant X_m_ is the largest eigenvalue of the system (7) at the steady state (*K_x_/β,K_y_*,0) (see [20]), and is denoted by 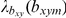 which takes the following form
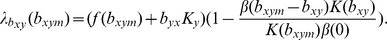



For a discussion of the notion of fitness see [21]. The invader's success will depend on its fitness in the following way: an invader with phenotypic characteristic *b_xym_* when initially rare will be able to invade the resident population with phenotypic characteristic *b_xy_* if 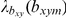
>0. Alternatively, if 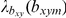
<0, the invading population will die out. A phenotypic value for which the local fitness gradient is zero is called an ‘evolutionarily singular strategy’ [21], in our case denoted by b^*^. According to [21] and [22], at a singular strategy several evolutionary outcomes are possible. A singular strategy can: lack convergence stability and therefore act as an evolutionary repellor; be both evolutionarily and convergence stable and therefore be the final outcome of the evolution (also called ‘continuously stable strategy’); and, finally, be convergence stable but not evolutionarily stable, in which case it is called a ‘branching point’. These classifications are based on the assumption that, away from a singular strategy, the principle of mutual exclusion holds so that, after a successful invasion, the nearby invading population takes over and replaces the resident population. However, in a small neighbourhood of a singular strategy, the successful invasion by a nearby mutant can, under certain conditions, result in the coexistence of the invader and of the resident type populations [22].

Here the outcome of the evolution of cooperation is investigated in a manner similar to the one described in [23]. The results are summarized in the Table 1 and detailed calculations are presented in Text S2.

**Table 1:** Possible evolutionary singularities (b^*^) with different functional forms of *K* and *β*.

	*β* concave near *0* *(β″(0)<0)*	*β* linear near *0* *(β″(0) = 0)*	*β* convex near *0* *(β″(0)>0)*
*K* concave near b^*^ (K″(b^*^)<0)	Branching point or CSS	CSS	CSS
*K* linear near b^*^ (K″(b^*^) = 0)	Branching point	Degenerate	CSS
*K* convex near b^*^ (K″(b^*^)>0)	Branching point or repellor	Branching point or repellor	Branching point; repellor or CSS

**Figure 5:**
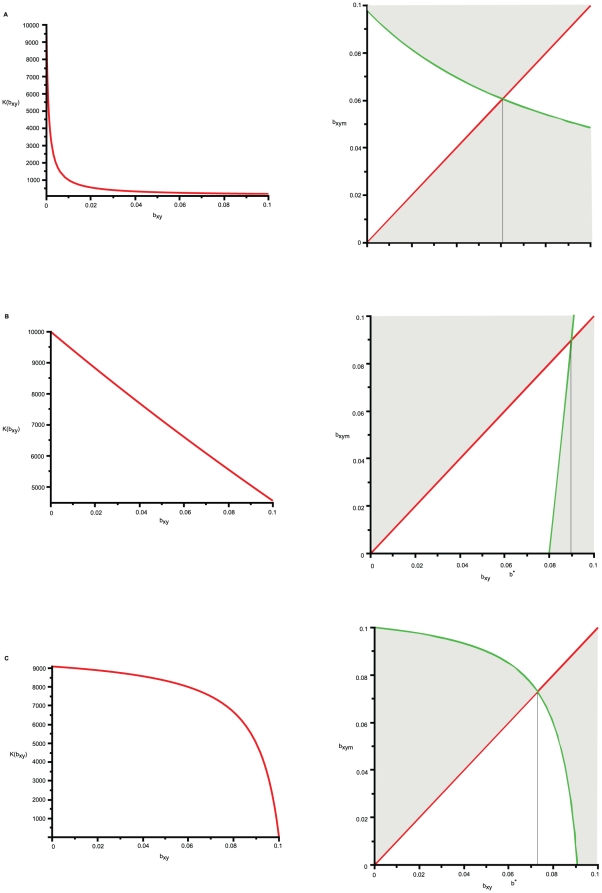
Evolutionary outcomes. The left hand side of the figure represents examples of different trade-offs while the right hand side of the figure gives the corresponding pairwise invisibility plots, PIPs, [22]. The shaded area indicate the combinations of *b_xy_* and *b_xym_* for which the fitness of the mutant, 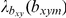, is positive. In all cases *f*(*x*) = 0.025−0.16*x*, β(*x*) = 1−(*x*+0.1)/0.2 while *K*(*x*) = α_1_(*x*−α_2_)/(*x*−α_3_). **A**. the left hand side shows a steep convex *K* function with α_1_ = 100, α_2_ = −0.1 and α_3_ = −0.001 with a corresponding PIP on the right hand side illustrating that in this case b^*^ is a repellor; **B**. the left hand side shows a shallow convex *K* function with α_1_ = −50000, α_2_ = 0.2 and α_3_ = −1 with a corresponding PIP on the right hand side illustrating that in this case b^*^ is a branching point; (C) the left hand side shows a concave *K* function with α_1_ = 10000, α_2_ = 0.1 and α_3_ = 0.11 with a corresponding PIP on the right hand side illustrating that in this case b^*^ is a CSS.

Under what conditions the cheating type X_m_ that does not invest into cooperation and hence have *b_xym_* = 0, outcompetes the resident type X that has a non-zero investment into cooperation namely *b_xy_*>0? From Table 1 it follows that this is only possible when *K* is a convex function near the singular strategy b^*^(see Figure 5A left for an example). In that case the singular strategy could be a repellor which means that if the benefit to Y of the resident population X, *b_xy_*, is less than b^*^, the system will evolve towards the population where there is no benefit to Y from X. On the other hand if *b_xy_*>b^*^, the system will evolve towards the population where Y receives a maximal possible benefit from X (Figure 5A right).

In all of the remaining cases the following outcomes are possible. The singular strategy b^*^ is a continuously stable strategy (CSS) which implies that an initially monomorphic population of type X with the trait *b_xy_* remains monomorphic throughout the course of evolution with a non-zero investment into cooperation, b^*^, representing the final outcome of evolution. Alternatively b^*^could be a branching point whereby an initially monomorphic population becomes dimorphic in the vicinity of b^*^. In this case the outcome of evolution is a population containing two or more phenotypes with varying degree of investment into cooperation.

Table 1 shows that convex *K* does not always imply that the singular strategy b^*^ is a repellor. Under certain conditions (see Text S2) it can also be a branching point (Figure 5B) or a CSS. Therefore the instances where a cheat phenotype with *b_xy_* = 0 outcompetes and replaces a cooperating phenotype with *b_xy_*>0 could be viewed as relatively rare.

However, given that the carrying capacity trade-off is motivated by the inhibitory properties of methionine [19] we argue that a concave K illustrated in Figure 5C left would be more appropriate as there is a threshold concentration of methionine above which the carrying capacity decreases. In this case the singular strategy is never a repellor and therefore cheats never outcompete and replace cross-feeding cooperators (Figure 5C right).

In this section we have classified a variety of evolutionary outcomes with respect to persistence of cooperation that depend on the shape of the *K* and ß*β* trade-offs. While there are many experimental evolutionary studies on microbial cooperation that have acknowledged the existence of different outcomes when a cooperative population is invaded by a mutant with a different investment into cooperation [24]–[29] very little is still known about the conditions that favour the evolution of cooperative cross-feeding between species. Pioneering work on the experimental evolution of novel cooperation between two cross-feeding species, [18], has been an important step towards a better understanding of the factors that enable interspecific cooperation in a cross-feeding interaction. But as highlighted by the author in [18] there is still “a lack of clear explanation of the mechanisms necessary for the evolutionary origin of cooperation, particularly between species”. Further experimental studies are needed to shed light on this important problem.

## Discussion

When the cost of cross-feeding to the donor (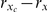) is greater or equal to the benefit to the recipient (*b_xy_*) cooperation is never favoured. Indeed, by definition a reciprocal interaction provides a direct fitness benefit to the cooperators and this suggests that a cooperative trait will only be selected if the benefit to cooperate is higher than its cost. Additionally, this also reflects the fact that an individual that doesn't pay the cost of cooperation in the short term will not gain the benefit of cooperation in the long term [4].

Previous theoretical results indicate that the cross-feeding is more easily selected when its cost to the donor is low per benefit to the recipient, in other words 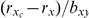 is sufficiently small [30], [31] and when the recipient already provides a large cross-feeding benefit to the donor, in other words when *b_xy_* is sufficiently large [17]. Our study recovers the same outcomes (Figure 1A,B) but in addition we obtain results that are at odds with those presented in [17] in the case of growth at intermediate densities. Before summarising the differences in outcomes we note that they come about due to the fact that while we study the non-linear system (1) the results in [17] are obtained using a linear approximation of (1). Contrary to [17], we find that the cross-feeder does not always outgrow the non cross-feeder when the benefit of cross-feeding to X per individual of type Y is *b_yx_>0* and *r_x_≤r_y_* (Figure 2B,C). In addition to [17] we find that steep trade-offs can also promote the evolution of cross-feeding (Figure 1C). Namely, a decrease in the intrinsic growth rates increases the range of values of 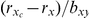 for which the cross-feeding is favoured. This is explained by the fact that when intrinsic growth rates are low compared to the benefit of cross-feeding, the cross-feeding term dominates the overall growth of microorganisms and therefore the cost of cross-feeding is not required to be too low for the cross-feeding to be favoured. Surprisingly, our model indicates that in some cases cross-feeding is favoured even if the cost to the donor is up to 80% of the value of the benefit to the recipient. This seems to suggest the following. Firstly, if populations have high intrinsic growth rate and are therefore less dependent on the cross-feeding interactions to grow, cross-feeding interactions are less favoured. Secondly, an increase in synergic benefit of cooperation should result in cooperation being more easily selected for [32].

The advantage of cross-feeding is also known to change with initial population densities of interacting microorganisms [17]. In addition we find that a reduction in the initial population densities can lead to a dramatic change in the outcome from cross-feeding being favoured at intermediate densities to cross-feeders never being able to outgrow non cross-feeders.

In evolutionary terms, our study reveals a result different to that reported in [17]. We find that once a population of two cross-feeders has been established in a spatially isolated colony, the large populations of cross-feeders are not vulnerable to small numbers of exploiting genotypes that arise through migration or mutation and who share in the cross-feeding resources but do not reciprocate in cross-feeding themselves. However, this result relies on the assumption that all microbial types have the same carrying capacity. Subsequently we considered a more general evolutionary model assuming that X and Y utilize different resources and therefore have different carrying capacities, [18]. Motivated by [19] we also introduced the following additional trade-offs: an increased investment into cooperation results in an increased competitive ability but a decreased carrying capacity. We find that an exploiting genotype that does not reciprocate in cross-feeding can take over and replace the resident cooperator genotype only in certain cases when the carrying capacity trade-off is convex. Given that such trade-off is motivated by the inhibitory properties of methionine [19] we argue that a concave trade-off illustrated in Figure 5C would be more appropriate as there is a threshold concentration of methionine above which the carrying capacity decreases. Our results indicate that a concave trade-off between investment into cooperation and carrying capacity is most likely to give rise to populations containing a single phenotype that has a non-zero investment into cooperation.

In conclusion our results have a number of important messages. Firstly, the shape of the trade-off between the cost and benefit of cooperation has a profound effect on the success of cross-feeders (cooperators) in comparison to non cross-feeders (cheats). In other words whether cross-feeding is favoured or not depends on whether the cost to the donor decreases slower or faster than the benefit to the recipient. This is in accordance with both classical [33]–[35] and recent [36]–[40] theoretical work showing that the precise form of the trade-off curves crucially determines the outcome of evolution. Therefore in order to deepen our understanding of the evolution of cooperative cross-feeding, it is extremely important to obtain precise estimation of the shape of the cost/benefit trade-off. Elucidating the shape of a trade-off relationship in general is something that has so far proven to be particularly challenging. However, due to their large population sizes, short generation times and known genetic structure microorganisms present an ideal system with which to experimentally study the nature and form of trade-off relationships [41], [42].

Secondly, we have demonstrated that the impact of the trade-off between the cost and the benefit of cross-feeding varies with different environments. For example, in the environments where the intrinsic growth rates of microbes under consideration are higher than the benefit of cross-feeding, cooperative behaviour is favoured only for sufficiently shallow trade-offs. However, in the environments where the intrinsic growth rates are lower than the benefit of cross-feeding, cooperation behaviour is favoured for a large range of trade-off slopes.

Finally, when considering the evolution of cross-feeding we found that if all interacting individuals have the same carrying capacity a small population of cheats could not invade an already established population of cooperating cross-feeders. If we assume that cross-feeding species specialize on different resources and hence have different carrying capacities the outcome of evolution depends on the shape of the trade-off between investment into cooperation and competitive ability and the trade-off between investment into cooperation and carrying capacity. The most common outcome of evolution is either polymorphism where the evolving population contains two or more genotypes with varying degree of cooperation or monomorphism where the evolving population contains a single phenotype that makes a non-zero investment into cooperation. This further demonstrates that cross-feeding could be viewed as a robust interaction, a result that accords with a large number of cross-feeding examples readily observed in nature.

## Materials and Methods

Numerical simulations were performed using MATLAB. Parameter values for each illustration are provided in the figure legends.

## Supporting Information

Text S1 Calculating the steady states of system (6) and their stability.
(0.17 MB PDF)

Text S2 Detailed calculations of the results presented in Table 1.
(0.19 MB PDF)
