# Supplementary material for: Evolution of Cooperative Cross-Feeding Could Be Less Challenging Than Originally Thought
Source: PLoS One. 2010 Nov 29;5(11):e14121. doi: 10.1371/journal.pone.0014121 (PMC2994712; doi:10.1371/journal.pone.0014121)
Supplement: Text S1 — Calculating the steady states of system (6) and their stability. (0.17 MB PDF) [file pone.0014121.s001.pdf]

Text S1. Calculating steady states of system (6) and their stability

Steady states of system (6) can be found by solving the following set of equations:

$$
\begin{aligned}
0 &= X(r_x + b_{yx}Y)(1 - \frac{\beta X}{K_x}),\\
0 &= Y(r_y + b_{xy}X)(1 - \frac{Y}{K_y}).
\end{aligned}
\qquad \text{(A1)}
$$

for X and Y. It is easy to see that there are four steady states $(X_1^*, Y_1^*)=(0,0)$, $(X_2^*, Y_2^*)=(K_x/\beta, 0)$, $(X_3^*, Y_3^*)=(0, K_y)$ and $(X_4^*, Y_4^*)=(K_x/\beta, K_y)$. The Jacobian matrix of (A1) takes the form

$$
J(X,Y) = \begin{pmatrix} (r_x + b_{yx}Y)(1 - \frac{2\beta X}{K_x}) & b_{yx}X(1 - \frac{\beta X}{K_x}) \\ b_{xy}Y(1 - \frac{Y}{K_y}) & (r_y + b_{xy}X)(1 - \frac{2Y}{K_y}) \end{pmatrix}
$$

evaluated at $(X,Y)=(X_i^*, Y_i^*)$ where $i=1..4$. For the trivial steady state we have

$$
J(0,0) = \begin{pmatrix} r_x & 0 \\ 0 & r_y \end{pmatrix},
$$

hence the trivial steady state is unstable. Similarly for the semi-trivial steady states we have

$$
J(\frac{K_x}{\beta},0) = \begin{pmatrix} -r_x & 0 \\ 0 & r_y + b_{xy}\frac{K_x}{\beta} \end{pmatrix} \text{ and } J(0,K_y) = \begin{pmatrix} r_x + b_{yx}K_y & 0 \\ 0 & -r_y \end{pmatrix},
$$

from which we conclude that both semi-trivial steady states are also unstable. Finally for the non-trivial steady state we have

$$
J(\frac{K_x}{\beta},0) = \begin{pmatrix} -(r_x + b_{yx}K_y & 0 \\ 0 & -(r_y + b_{xy}\frac{K_x}{\beta}) \end{pmatrix},
$$

and therefore the non-trivial steady state is stable.
