# Supplementary material for: Evolution of Cooperative Cross-Feeding Could Be Less Challenging Than Originally Thought
Source: PLoS One. 2010 Nov 29;5(11):e14121. doi: 10.1371/journal.pone.0014121 (PMC2994712; doi:10.1371/journal.pone.0014121)
Supplement: Text S2 — Detailed calculations of the results presented in Table 1. (0.19 MB PDF) [file pone.0014121.s002.pdf]

Text S2. Detailed calculations of the results presented in Table 1.

Consider the rare mutant strategy X_m in the resident population X. The mutant increases in number of its growth rate

$$\lambda_{b_{xy}}(b_{xym}) = (f(b_{xym}) + b_{yx}K_y)(1 - \frac{\beta(b_{xym} - b_{xy})K(b_{xy})}{K(b_{xym})\beta(0)})$$

is positive (see system of equations 7) while a mutant with negative growth rate dies out (see [22, 23] for details). The resident population has zero growth rate ($\lambda_{b_{xy}}(b_{xy}) = 0$) at equilibrium population density $X(b_{xy})=K(b_{xy})/\beta(0)$. It follows that a mutant strategy b_{xym} that is slightly larger than b_{xy} can invade and replace the resident if the fitness gradient

$$\left.\frac{\partial\lambda_{b_{xy}}(b_{xym})}{\partial b_{xym}}\right|_{b_{xym}=b_{xy}} = (f(b_{xy}) + b_{yx}K_y)(\frac{K'(b_{xy})}{K(b_{xy})} - \frac{\beta'(0)}{\beta(0)})$$

is positive; mutants with $b_{xym} < b_{xy}$ can invade if the fitness gradient is negative. Repeated invasions and substitutions result in directional evolution until the population reaches an evolutionary singularity where the fitness gradient is zero. The singular strategy b^* can subsequently be classified in the following way. According to Geritz et al 1998 if

$$\left[\frac{\partial^2\lambda_{b_{xy}}(b_{xym})}{\partial b_{xym}{}^2}\right]_{b_{xym}=b_{xy}=b^*} = (f(b^*) + b_{yx}K_y)(-\frac{\beta''(0)}{\beta(0)} + \frac{K''(b^*)}{K(b^*)})<0 \quad \text{(B1)}$$

the singular strategy is evolutionary stable. If

$$\frac{d}{db_{xy}}\left[\left.\frac{\partial\lambda_{b_{xy}}(b_{xym})}{\partial b_{xym}}\right|_{b_{xym}=b_{xy}}\right]_{b^*} = (f(b^* + b_{yx}K_y)(\frac{K''(b^*)}{K(b^*)} - \left(\frac{K'(b^*)}{K(b^*)}\right)^2)<0 \quad \text{(B2)}$$

the singular strategy is convergence stable. Therefore if (B1) and (B2) hold the singular strategy is a continuously stable strategy (CSS); if (B2) holds but (B1) does not so that

$$-\frac{\beta''(0)}{\beta(0)} + \frac{K''(b^*)}{K(b^*)} > 0 \quad \text{(B3)}$$

the singular strategy is an evolutionary branching point. Finally if (B2) does not hold so that

$$\frac{K''(b^*)}{K(b^*)} - \left(\frac{K'(b^*)}{K(b^*)}\right)^2 > 0 \quad \text{(B4)}$$

the singular strategy is a repellor.
